# 1,2-Bis(undecyl­sulfan­yl)benzene

**DOI:** 10.1107/S1600536808014712

**Published:** 2008-05-21

**Authors:** Etsuko Tomiyama, Kazuo Miyamura

**Affiliations:** a Department of Chemistry, Faculty of Science, Tokyo University of Science, Kagurazaka 1-3, Shinjuku-ku, Tokyo 162-8601, Japan

## Abstract

In the title compound, C_28_H_50_S_2_, the alkyl chains adopt a fully extended all-*trans* conformation and each of them is almost perfectly planar. One of the alkyl chains is coplanar with the benzene ring and the other is twisted out of the benzene ring plane; the C—C—S—C torsion angles are 176.4 (2) and 80.8 (3)°. In the crystal structure, an inter­molecular S⋯S inter­action [3.2123 (13) Å] links the mol­ecules into a centrosymmetric dimer; dimers are linked through weak C—H⋯π and C—H⋯S inter­actions, forming a column along the *a* axis.

## Related literature

For related literature, see: Alves *et al.* (2004 ▶); Huynh *et al.* (2002 ▶); Liu *et al.* (2007 ▶); Robertson & Cronin (2002 ▶); Salvatore *et al.* (2005 ▶); Tomiyama *et al.* (2007 ▶).
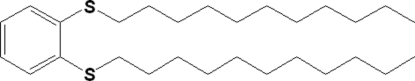


## Experimental

### 

#### Crystal data

C_28_H_50_S_2_
*M* *_r_* = 450.80
Monoclinic, 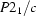

*a* = 5.4024 (10) Å
*b* = 16.863 (3) Å
*c* = 29.611 (5) Å
β = 91.245 (3)°
*V* = 2696.9 (8) Å^3^
*Z* = 4
Mo *K*α radiation
μ = 0.21 mm^−1^
*T* = 100 (1) K
0.55 × 0.11 × 0.10 mm

#### Data collection

Bruker SMART APEX CCD-detector diffractometer
Absorption correction: analytical (*XPREP*; Bruker 2000 ▶) *T*_min_ = 0.901, *T*_max_ = 0.991
15683 measured reflections
5958 independent reflections
4452 reflections with *I* > 2σ(*I*)
*R*_int_ = 0.086

#### Refinement

*R*[*F* ^2^ > 2σ(*F* ^2^)] = 0.087
*wR*(*F* ^2^) = 0.205
*S* = 1.14
5958 reflections
273 parameters
H-atom parameters constrained
Δρ_max_ = 0.75 e Å^−3^
Δρ_min_ = −0.55 e Å^−3^

### 

Data collection: *SMART* (Bruker, 2001 ▶); cell refinement: *SAINT* (Bruker, 2001 ▶); data reduction: *SAINT*; program(s) used to solve structure: *SHELXS97* (Sheldrick, 2008 ▶); program(s) used to refine structure: *SHELXL97* (Sheldrick, 2008 ▶); molecular graphics: *ORTEPIII* (Burnett & Johnson, 1996 ▶) and *Mercury* (Macrae *et al.*, 2006 ▶); software used to prepare material for publication: *SHELXL97*, *KENX* (Sakai, 2002 ▶), *ORTEPIII* and *Mercury*.

## Supplementary Material

Crystal structure: contains datablocks global, I. DOI: 10.1107/S1600536808014712/ci2597sup1.cif

Structure factors: contains datablocks I. DOI: 10.1107/S1600536808014712/ci2597Isup2.hkl

Additional supplementary materials:  crystallographic information; 3D view; checkCIF report

## Figures and Tables

**Table 1:** Hydrogen-bond geometry (Å, °)

*D*—H⋯*A*	*D*—H	H⋯*A*	*D*⋯*A*	*D*—H⋯*A*
C18—H18*B*⋯S1^i^	0.99	2.99	3.749 (4)	134
C7—H7*A*⋯*Cg*^ii^	0.99	2.67	3.567 (16)	151

Symmetry codes: (i) 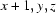; (ii) 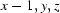. *Cg* is the centroid of the C1–C6 ring.

**Fig. 1.**
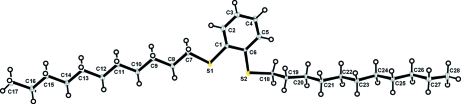
An ORTEPIII (Burnett & Johnson, 1996) view of the title compound, showing 50% probability displacement ellipsoids and the atom-numbering scheme.

**Fig. 2.**
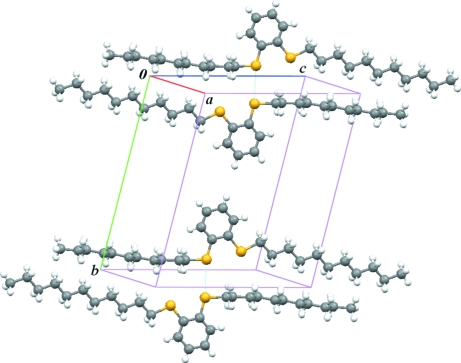
A partial packing diagram of the title compound, showing symmetrically paired molecules. S···S interactions are shown by dashed lines.

## supplementary crystallographic information

### Comment

Thioethers have emerged as preeminent classes of organic compounds, which hold useful applications as key reagents in organic synthesis, bio-organic, mechanical, and heterocyclic chemistry. The coordination chemistry of dithiolate ligands has also been extensively studied (Liu *et al.*, 2007; Alves *et al.*, 2004; Huynh *et al.*, 2002). In recent years, transition metal bis(dithiolene) complexes, with square-planar coordination geometry, have been used widely as building blocks for conducting and magnetic materials (Robertson & Cronin, 2002). We previously reported the crystal structure of a dithiolate complex salt (Tomiyama *et al.*, 2007). In order to explore crystal structures of new dithiole compounds and to gain more insight into the structure-regulating ability of intermolecular S···S, C—H···S  interactions, the title compound was synthesized and its structure was analysized by X-ray analysis.

The structure of the title molecule is shown in Fig. 1. The alkyl chains are in the fully extended all-trans conformation and each alkyl chain is almost perfectly planar. The C8—C7—S1—C1 and C19—C18—S2—C6 torsion angles of 176.4 (2)° and 80.8 (3)°, respectively, indicate that non-hydrogen atoms of one of the side chains is coplanar with the benzene ring, and the other chain is twisted out of the benzene plane.

In the crystal structure, an intermolecular S···S interaction [S2···S2(1-x,-y,1-z) = 3.2123 (13) Å] shorter than 3.70 Å, the sum of van der Waals radii, links the molecules into a centrosymmetric dimer (Fig. 2). The dimers are linked through weak C—H···π (between C7—H7A and benzene ring) and C—H···S interactions (Table 1) to form a column along the *a* axis.

### Experimental

All starting materials were of reagent grade and used without further purification. 1,2-Bis(undecylthio)benzene was prepared by a literature procedure (Salvatore *et al.*, 2005): Benzene-1,2-dithiol (1 mmol) was stirred under argon atmosphere at temperature for 1 h in the presence of caesium carbonate (2.2 mmol), tetrabutylammonium iodide (TBAI; 2.2 mmol) and anhydrous DMF. The reaction mixture was subsequently cooled to 273 K, added with undecylbromide (2.2 mmol), and the reaction mixture stirred for 2 h and then allowed to return to room temperature. The title compound was obtained by slow evaporation method from the reaction mixture at room temperature. White needle-shaped crystals of suitable size for X-ray diffraction were obtained. ^1^H NMR (CDCl_3_): δ 0.88 (t, *J* = 6.7, 6H), 1.26–1.44 (m, 32H), 1.66 (quin, *J* = 7.3, 4H), 2.90 (t, *J* = 7.4, 4H), 7.11–7.14 (m, 2H), 7.24–7.27 (m, 2H). ^13^C NMR (CDCl_3_): δ 14.1, 22.7, 28.8, 29.0, 29.2, 29.3, 29.5, 29.6, 31.9, 33.2, 125.9, 128.5, 137.2.

### Refinement

All H atoms were positioned geometrically and allowed to ride on their attached atoms, with C-H = 0.95–0.99 Å and *U*_iso_ = 1.2*U*_eq_(C) or 1.5*U*_eq_(methyl C).

### Crystal data

**Table:**

C_28_H_50_S_2_	*F*_000_ = 1000
*M**_r_* = 450.80	*D*_x_ = 1.110 Mg m^−^^3^
Monoclinic, *P*2_1_/*c*	Mo *K*α radiation λ = 0.71073 Å
Hall symbol: -P 2ybc	Cell parameters from 3006 reflections
*a* = 5.4024 (10) Å	θ = 2.4–27.7º
*b* = 16.863 (3) Å	µ = 0.21 mm^−^^1^
*c* = 29.611 (5) Å	*T* = 100 (1) K
β = 91.245 (3)º	Needle, white
*V* = 2696.9 (8) Å^3^	0.55 × 0.11 × 0.10 mm
*Z* = 4	

### Data collection

**Table:**

Bruker SMART APEX CCD-detector diffractometer	5958 independent reflections
Radiation source: fine-focus sealed tube	4452 reflections with *I* > 2σ(*I*)
Monochromator: graphite	*R*_int_ = 0.086
Detector resolution: 8.366 pixels mm^-1^	θ_max_ = 27.5º
*T* = 100(1) K	θ_min_ = 3.0º
ω scans	*h* = −6→5
Absorption correction: analytical(XPREP; Bruker 2000)	*k* = −21→21
*T*_min_ = 0.901, *T*_max_ = 0.991	*l* = −24→37
15683 measured reflections	

### Refinement

**Table:**

Refinement on *F*^2^	Secondary atom site location: difference Fourier map
Least-squares matrix: full	Hydrogen site location: inferred from neighbouring sites
*R*[*F*^2^ > 2σ(*F*^2^)] = 0.088	H-atom parameters constrained
*wR*(*F*^2^) = 0.205	*w* = 1/[σ^2^(*F*_o_^2^) + (0.0976*P*)^2^] where *P* = (*F*_o_^2^ + 2*F*_c_^2^)/3
*S* = 1.14	(Δ/σ)_max_ = 0.001
5958 reflections	Δρ_max_ = 0.75 e Å^−^^3^
273 parameters	Δρ_min_ = −0.55 e Å^−^^3^
Primary atom site location: structure-invariant direct methods	Extinction correction: none

### Special details

**Table:**

Experimental. The first 50 frames were rescanned at the end of data collection to evaluate any possible decay phenomenon. Since it was judged to be negligible, no decay correction was applied to the data.
Geometry. All e.s.d.'s (except the e.s.d. in the dihedral angle between two l.s. planes) are estimated using the full covariance matrix. The cell e.s.d.'s are taken into account individually in the estimation of e.s.d.'s in distances, angles and torsion angles; correlations between e.s.d.'s in cell parameters are only used when they are defined by crystal symmetry. An approximate (isotropic) treatment of cell e.s.d.'s is used for estimating e.s.d.'s involving l.s. planes.Mean-plane data from final *SHELXL* refinement run:-Least-squares planes (*x*,*y*,*z* in crystal coordinates) and deviations from them (* indicates atom used to define plane)- 3.2338 (0.0068) *x* - 0.2627 (0.0262) *y* + 24.0951 (0.0272) *z* = 10.3067 (0.0188)* -0.0131 (0.0025) C1 * 0.0083 (0.0027) C2 * 0.0004 (0.0029) C3 * -0.0044 (0.0028) C4 * -0.0004 (0.0026) C5 * 0.0092 (0.0026) C6Rms deviation of fitted atoms = 0.00761.5719 (0.0210) *x* + 16.1263 (0.0188) *y* + 0.6605 (0.0846) *z* = 2.7127 (0.0343)Angle to previous plane (with approximate e.s.d.) = 80.44 (0.21)* 0.0000 (0.0001) S2 * 0.0000 (0.0000) C18 * 0.0000 (0.0000) C19Rms deviation of fitted atoms = 0.0000
Refinement. Refinement of *F*^2^ against ALL reflections. The weighted *R*-factor *wR* and goodness of fit *S* are based on *F*^2^, conventional *R*-factors *R* are based on *F*, with *F* set to zero for negative *F*^2^. The threshold expression of *F*^2^ > σ(*F*^2^) is used only for calculating *R*-factors(gt) *etc*. and is not relevant to the choice of reflections for refinement. *R*-factors based on *F*^2^ are statistically about twice as large as those based on *F*, and *R*- factors based on ALL data will be even larger.

### Fractional atomic coordinates and isotropic or equivalent isotropic displacement parameters (Å^2^)

**Table:**

	*x*	*y*	*z*	*U*_iso_*/*U*_eq_	
C1	0.3636 (6)	0.23339 (18)	0.47850 (11)	0.0143 (7)	
C2	0.3445 (6)	0.31584 (19)	0.47775 (11)	0.0177 (7)	
H2	0.2129	0.3404	0.4611	0.021*	
C3	0.5152 (6)	0.36204 (19)	0.50091 (12)	0.0206 (8)	
H3	0.5028	0.4182	0.4998	0.025*	
C4	0.7044 (6)	0.3267 (2)	0.52580 (11)	0.0196 (7)	
H4	0.8218	0.3588	0.5417	0.024*	
C5	0.7236 (6)	0.24514 (19)	0.52766 (11)	0.0172 (7)	
H5	0.8540	0.2213	0.5449	0.021*	
C6	0.5529 (6)	0.19786 (18)	0.50448 (10)	0.0126 (6)	
C7	−0.0418 (6)	0.23663 (19)	0.41612 (11)	0.0161 (7)	
H7A	−0.1406	0.2689	0.4370	0.019*	
H7B	0.0570	0.2729	0.3973	0.019*	
C8	−0.2111 (6)	0.18638 (19)	0.38639 (11)	0.0156 (7)	
H8A	−0.3070	0.1502	0.4057	0.019*	
H8B	−0.1091	0.1534	0.3663	0.019*	
C9	−0.3894 (6)	0.23563 (19)	0.35769 (11)	0.0160 (7)	
H9A	−0.4946	0.2674	0.3778	0.019*	
H9B	−0.2933	0.2730	0.3391	0.019*	
C10	−0.5543 (6)	0.18594 (19)	0.32679 (11)	0.0166 (7)	
H10A	−0.6531	0.1496	0.3455	0.020*	
H10B	−0.4486	0.1531	0.3073	0.020*	
C11	−0.7305 (6)	0.23463 (19)	0.29673 (11)	0.0165 (7)	
H11A	−0.8384	0.2668	0.3162	0.020*	
H11B	−0.6320	0.2716	0.2784	0.020*	
C12	−0.8924 (6)	0.1844 (2)	0.26526 (11)	0.0175 (7)	
H12A	−0.7845	0.1524	0.2458	0.021*	
H12B	−0.9904	0.1473	0.2836	0.021*	
C13	−1.0683 (6)	0.23277 (19)	0.23545 (11)	0.0171 (7)	
H13A	−1.1768	0.2645	0.2550	0.021*	
H13B	−0.9702	0.2702	0.2173	0.021*	
C14	−1.2293 (6)	0.18315 (19)	0.20373 (11)	0.0167 (7)	
H14A	−1.3252	0.1451	0.2219	0.020*	
H14B	−1.1208	0.1521	0.1838	0.020*	
C15	−1.4082 (6)	0.2314 (2)	0.17450 (11)	0.0174 (7)	
H15A	−1.3129	0.2701	0.1567	0.021*	
H15B	−1.5193	0.2616	0.1943	0.021*	
C16	−1.5645 (6)	0.1807 (2)	0.14234 (11)	0.0203 (8)	
H16A	−1.6564	0.1411	0.1601	0.024*	
H16B	−1.4533	0.1514	0.1221	0.024*	
C17	−1.7491 (7)	0.2284 (2)	0.11360 (12)	0.0247 (8)	
H17A	−1.8648	0.2558	0.1333	0.037*	
H17B	−1.8413	0.1926	0.0933	0.037*	
H17C	−1.6598	0.2675	0.0957	0.037*	
C18	0.8229 (6)	0.06584 (19)	0.53962 (10)	0.0147 (7)	
H18A	0.8749	0.0117	0.5311	0.018*	
H18B	0.9610	0.1021	0.5327	0.018*	
C19	0.7839 (6)	0.06751 (19)	0.58997 (11)	0.0151 (7)	
H19A	0.6565	0.0280	0.5979	0.018*	
H19B	0.7241	0.1206	0.5990	0.018*	
C20	1.0263 (6)	0.04874 (19)	0.61545 (11)	0.0169 (7)	
H20A	1.0864	−0.0037	0.6055	0.020*	
H20B	1.1518	0.0886	0.6071	0.020*	
C21	1.0057 (6)	0.04781 (19)	0.66646 (11)	0.0182 (7)	
H21A	0.9020	0.0022	0.6753	0.022*	
H21B	0.9214	0.0969	0.6762	0.022*	
C22	1.2558 (6)	0.0419 (2)	0.69080 (11)	0.0172 (7)	
H22A	1.3383	−0.0074	0.6811	0.021*	
H22B	1.3597	0.0870	0.6813	0.021*	
C23	1.2446 (6)	0.04204 (19)	0.74200 (11)	0.0190 (7)	
H23A	1.1481	−0.0046	0.7517	0.023*	
H23B	1.1555	0.0902	0.7518	0.023*	
C24	1.4977 (6)	0.0400 (2)	0.76552 (11)	0.0182 (7)	
H24A	1.5864	−0.0082	0.7558	0.022*	
H24B	1.5943	0.0865	0.7556	0.022*	
C25	1.4887 (6)	0.0404 (2)	0.81672 (11)	0.0188 (7)	
H25A	1.3865	−0.0049	0.8266	0.023*	
H25B	1.4065	0.0897	0.8266	0.023*	
C26	1.7422 (6)	0.0350 (2)	0.83994 (11)	0.0194 (7)	
H26A	1.8269	−0.0133	0.8292	0.023*	
H26B	1.8421	0.0813	0.8309	0.023*	
C27	1.7338 (7)	0.0323 (2)	0.89105 (12)	0.0243 (8)	
H27A	1.6665	0.0831	0.9022	0.029*	
H27B	1.6197	−0.0105	0.9001	0.029*	
C28	1.9854 (7)	0.0181 (2)	0.91318 (13)	0.0298 (9)	
H28A	2.0508	−0.0330	0.9031	0.045*	
H28B	1.9693	0.0176	0.9461	0.045*	
H28C	2.0989	0.0606	0.9046	0.045*	
S1	0.16106 (14)	0.17012 (5)	0.44775 (3)	0.0151 (2)	
S2	0.55814 (15)	0.09312 (5)	0.50441 (3)	0.0171 (2)	

### Atomic displacement parameters (Å^2^)

**Table:**

	*U*^11^	*U*^22^	*U*^33^	*U*^12^	*U*^13^	*U*^23^
C1	0.0147 (16)	0.0167 (16)	0.0114 (17)	−0.0035 (13)	−0.0017 (12)	0.0033 (13)
C2	0.0188 (17)	0.0186 (17)	0.0156 (18)	0.0018 (14)	−0.0028 (13)	0.0007 (14)
C3	0.0254 (18)	0.0135 (17)	0.0228 (19)	0.0004 (14)	−0.0050 (14)	−0.0015 (14)
C4	0.0227 (17)	0.0221 (18)	0.0137 (17)	−0.0030 (14)	−0.0066 (13)	−0.0013 (14)
C5	0.0180 (17)	0.0187 (17)	0.0149 (17)	0.0012 (13)	−0.0027 (13)	0.0018 (13)
C6	0.0147 (15)	0.0123 (15)	0.0109 (16)	−0.0034 (12)	−0.0017 (12)	0.0021 (12)
C7	0.0131 (16)	0.0191 (17)	0.0160 (18)	0.0021 (13)	−0.0040 (12)	0.0025 (13)
C8	0.0166 (16)	0.0161 (16)	0.0139 (17)	0.0007 (13)	−0.0045 (13)	−0.0011 (13)
C9	0.0153 (16)	0.0156 (16)	0.0169 (18)	0.0011 (13)	−0.0027 (13)	0.0024 (13)
C10	0.0137 (16)	0.0195 (17)	0.0163 (18)	0.0010 (13)	−0.0050 (13)	−0.0014 (13)
C11	0.0141 (16)	0.0189 (17)	0.0163 (18)	−0.0008 (13)	−0.0022 (13)	0.0003 (13)
C12	0.0157 (16)	0.0210 (18)	0.0157 (18)	−0.0016 (13)	−0.0041 (13)	0.0011 (14)
C13	0.0144 (16)	0.0204 (17)	0.0164 (18)	−0.0026 (13)	−0.0041 (13)	0.0013 (14)
C14	0.0155 (16)	0.0190 (17)	0.0155 (18)	−0.0002 (13)	−0.0038 (13)	0.0006 (13)
C15	0.0155 (16)	0.0211 (17)	0.0153 (18)	−0.0014 (13)	−0.0058 (13)	0.0030 (13)
C16	0.0206 (17)	0.0234 (18)	0.0165 (18)	−0.0009 (14)	−0.0083 (14)	0.0005 (14)
C17	0.0254 (19)	0.029 (2)	0.0190 (19)	0.0005 (15)	−0.0125 (15)	0.0036 (15)
C18	0.0125 (15)	0.0175 (16)	0.0139 (17)	−0.0016 (13)	−0.0055 (12)	0.0001 (13)
C19	0.0159 (16)	0.0152 (16)	0.0141 (17)	0.0024 (13)	−0.0033 (12)	0.0016 (13)
C20	0.0173 (16)	0.0176 (17)	0.0157 (18)	0.0003 (13)	−0.0054 (13)	−0.0002 (13)
C21	0.0222 (17)	0.0167 (17)	0.0154 (18)	0.0014 (14)	−0.0043 (14)	0.0030 (13)
C22	0.0204 (17)	0.0181 (17)	0.0128 (17)	0.0009 (13)	−0.0070 (13)	−0.0017 (13)
C23	0.0228 (18)	0.0155 (16)	0.0184 (19)	0.0007 (14)	−0.0061 (14)	0.0017 (13)
C24	0.0208 (17)	0.0181 (17)	0.0153 (18)	0.0010 (14)	−0.0071 (13)	0.0001 (13)
C25	0.0218 (18)	0.0183 (17)	0.0160 (18)	0.0014 (14)	−0.0052 (14)	0.0020 (13)
C26	0.0244 (18)	0.0154 (16)	0.0180 (19)	0.0018 (14)	−0.0090 (14)	−0.0032 (14)
C27	0.030 (2)	0.0217 (18)	0.020 (2)	0.0010 (15)	−0.0054 (15)	0.0003 (15)
C28	0.039 (2)	0.029 (2)	0.020 (2)	0.0013 (17)	−0.0158 (17)	0.0016 (16)
S1	0.0137 (4)	0.0177 (4)	0.0135 (4)	−0.0001 (3)	−0.0076 (3)	0.0013 (3)
S2	0.0189 (4)	0.0147 (4)	0.0173 (5)	−0.0011 (3)	−0.0110 (3)	0.0021 (3)

### Geometric parameters (Å, °)

**Table:**

C1—C2	1.394 (4)	C16—C17	1.526 (4)
C1—C6	1.401 (4)	C16—H16A	0.99
C1—S1	1.767 (3)	C16—H16B	0.99
C2—C3	1.379 (4)	C17—H17A	0.98
C2—H2	0.95	C17—H17B	0.98
C3—C4	1.382 (4)	C17—H17C	0.98
C3—H3	0.95	C18—C19	1.511 (4)
C4—C5	1.381 (4)	C18—S2	1.811 (3)
C4—H4	0.95	C18—H18A	0.99
C5—C6	1.389 (4)	C18—H18B	0.99
C5—H5	0.95	C19—C20	1.530 (4)
C6—S2	1.766 (3)	C19—H19A	0.99
C7—C8	1.515 (4)	C19—H19B	0.99
C7—S1	1.814 (3)	C20—C21	1.517 (4)
C7—H7A	0.99	C20—H20A	0.99
C7—H7B	0.99	C20—H20B	0.99
C8—C9	1.518 (4)	C21—C22	1.521 (4)
C8—H8A	0.99	C21—H21A	0.99
C8—H8B	0.99	C21—H21B	0.99
C9—C10	1.516 (4)	C22—C23	1.519 (5)
C9—H9A	0.99	C22—H22A	0.99
C9—H9B	0.99	C22—H22B	0.99
C10—C11	1.528 (4)	C23—C24	1.522 (4)
C10—H10A	0.99	C23—H23A	0.99
C10—H10B	0.99	C23—H23B	0.99
C11—C12	1.521 (4)	C24—C25	1.518 (4)
C11—H11A	0.99	C24—H24A	0.99
C11—H11B	0.99	C24—H24B	0.99
C12—C13	1.520 (4)	C25—C26	1.521 (4)
C12—H12A	0.99	C25—H25A	0.99
C12—H12B	0.99	C25—H25B	0.99
C13—C14	1.517 (4)	C26—C27	1.516 (5)
C13—H13A	0.99	C26—H26A	0.99
C13—H13B	0.99	C26—H26B	0.99
C14—C15	1.519 (4)	C27—C28	1.516 (5)
C14—H14A	0.99	C27—H27A	0.99
C14—H14B	0.99	C27—H27B	0.99
C15—C16	1.522 (4)	C28—H28A	0.98
C15—H15A	0.99	C28—H28B	0.98
C15—H15B	0.99	C28—H28C	0.98
			
S2···S2^i^	3.2134 (18)		
			
C2—C1—C6	119.2 (3)	C17—C16—H16B	108.9
C2—C1—S1	123.3 (2)	H16A—C16—H16B	107.7
C6—C1—S1	117.5 (2)	C16—C17—H17A	109.5
C3—C2—C1	120.5 (3)	C16—C17—H17B	109.5
C3—C2—H2	119.8	H17A—C17—H17B	109.5
C1—C2—H2	119.8	C16—C17—H17C	109.5
C2—C3—C4	120.1 (3)	H17A—C17—H17C	109.5
C2—C3—H3	120.0	H17B—C17—H17C	109.5
C4—C3—H3	120.0	C19—C18—S2	116.0 (2)
C5—C4—C3	120.3 (3)	C19—C18—H18A	108.3
C5—C4—H4	119.9	S2—C18—H18A	108.3
C3—C4—H4	119.9	C19—C18—H18B	108.3
C4—C5—C6	120.3 (3)	S2—C18—H18B	108.3
C4—C5—H5	119.9	H18A—C18—H18B	107.4
C6—C5—H5	119.9	C18—C19—C20	110.3 (3)
C5—C6—C1	119.6 (3)	C18—C19—H19A	109.6
C5—C6—S2	124.3 (2)	C20—C19—H19A	109.6
C1—C6—S2	116.0 (2)	C18—C19—H19B	109.6
C8—C7—S1	107.7 (2)	C20—C19—H19B	109.6
C8—C7—H7A	110.2	H19A—C19—H19B	108.1
S1—C7—H7A	110.2	C21—C20—C19	114.3 (3)
C8—C7—H7B	110.2	C21—C20—H20A	108.7
S1—C7—H7B	110.2	C19—C20—H20A	108.7
H7A—C7—H7B	108.5	C21—C20—H20B	108.7
C7—C8—C9	112.8 (3)	C19—C20—H20B	108.7
C7—C8—H8A	109.0	H20A—C20—H20B	107.6
C9—C8—H8A	109.0	C20—C21—C22	112.9 (3)
C7—C8—H8B	109.0	C20—C21—H21A	109.0
C9—C8—H8B	109.0	C22—C21—H21A	109.0
H8A—C8—H8B	107.8	C20—C21—H21B	109.0
C10—C9—C8	113.1 (3)	C22—C21—H21B	109.0
C10—C9—H9A	108.9	H21A—C21—H21B	107.8
C8—C9—H9A	108.9	C23—C22—C21	114.7 (3)
C10—C9—H9B	108.9	C23—C22—H22A	108.6
C8—C9—H9B	108.9	C21—C22—H22A	108.6
H9A—C9—H9B	107.8	C23—C22—H22B	108.6
C9—C10—C11	113.9 (3)	C21—C22—H22B	108.6
C9—C10—H10A	108.8	H22A—C22—H22B	107.6
C11—C10—H10A	108.8	C22—C23—C24	113.7 (3)
C9—C10—H10B	108.8	C22—C23—H23A	108.8
C11—C10—H10B	108.8	C24—C23—H23A	108.8
H10A—C10—H10B	107.7	C22—C23—H23B	108.8
C12—C11—C10	113.6 (3)	C24—C23—H23B	108.8
C12—C11—H11A	108.8	H23A—C23—H23B	107.7
C10—C11—H11A	108.8	C25—C24—C23	114.1 (3)
C12—C11—H11B	108.8	C25—C24—H24A	108.7
C10—C11—H11B	108.8	C23—C24—H24A	108.7
H11A—C11—H11B	107.7	C25—C24—H24B	108.7
C13—C12—C11	113.7 (3)	C23—C24—H24B	108.7
C13—C12—H12A	108.8	H24A—C24—H24B	107.6
C11—C12—H12A	108.8	C24—C25—C26	113.8 (3)
C13—C12—H12B	108.8	C24—C25—H25A	108.8
C11—C12—H12B	108.8	C26—C25—H25A	108.8
H12A—C12—H12B	107.7	C24—C25—H25B	108.8
C14—C13—C12	114.0 (3)	C26—C25—H25B	108.8
C14—C13—H13A	108.8	H25A—C25—H25B	107.7
C12—C13—H13A	108.8	C27—C26—C25	114.0 (3)
C14—C13—H13B	108.8	C27—C26—H26A	108.8
C12—C13—H13B	108.8	C25—C26—H26A	108.8
H13A—C13—H13B	107.7	C27—C26—H26B	108.8
C13—C14—C15	114.0 (3)	C25—C26—H26B	108.8
C13—C14—H14A	108.8	H26A—C26—H26B	107.6
C15—C14—H14A	108.8	C28—C27—C26	113.0 (3)
C13—C14—H14B	108.8	C28—C27—H27A	109.0
C15—C14—H14B	108.8	C26—C27—H27A	109.0
H14A—C14—H14B	107.7	C28—C27—H27B	109.0
C14—C15—C16	113.2 (3)	C26—C27—H27B	109.0
C14—C15—H15A	108.9	H27A—C27—H27B	107.8
C16—C15—H15A	108.9	C27—C28—H28A	109.5
C14—C15—H15B	108.9	C27—C28—H28B	109.5
C16—C15—H15B	108.9	H28A—C28—H28B	109.5
H15A—C15—H15B	107.8	C27—C28—H28C	109.5
C15—C16—C17	113.6 (3)	H28A—C28—H28C	109.5
C15—C16—H16A	108.9	H28B—C28—H28C	109.5
C17—C16—H16A	108.9	C1—S1—C7	104.66 (15)
C15—C16—H16B	108.9	C6—S2—C18	105.42 (15)
			
C6—C1—C2—C3	2.4 (5)	C13—C14—C15—C16	179.0 (3)
S1—C1—C2—C3	−177.1 (3)	C14—C15—C16—C17	178.6 (3)
C1—C2—C3—C4	−1.2 (5)	S2—C18—C19—C20	−176.2 (2)
C2—C3—C4—C5	−0.1 (5)	C18—C19—C20—C21	−179.2 (3)
C3—C4—C5—C6	0.1 (5)	C19—C20—C21—C22	−171.0 (3)
C4—C5—C6—C1	1.2 (5)	C20—C21—C22—C23	179.1 (3)
C4—C5—C6—S2	−179.9 (3)	C21—C22—C23—C24	−177.3 (3)
C2—C1—C6—C5	−2.4 (5)	C22—C23—C24—C25	179.8 (3)
S1—C1—C6—C5	177.1 (3)	C23—C24—C25—C26	177.6 (3)
C2—C1—C6—S2	178.6 (3)	C24—C25—C26—C27	−177.9 (3)
S1—C1—C6—S2	−1.9 (4)	C25—C26—C27—C28	173.9 (3)
S1—C7—C8—C9	−179.5 (2)	C2—C1—S1—C7	3.0 (3)
C7—C8—C9—C10	178.3 (3)	C6—C1—S1—C7	−176.4 (3)
C8—C9—C10—C11	−178.5 (3)	C8—C7—S1—C1	176.4 (2)
C9—C10—C11—C12	179.0 (3)	C5—C6—S2—C18	0.0 (3)
C10—C11—C12—C13	179.8 (3)	C1—C6—S2—C18	179.0 (3)
C11—C12—C13—C14	179.6 (3)	C19—C18—S2—C6	80.8 (3)
C12—C13—C14—C15	179.0 (3)		

Symmetry codes: (i) −*x*+1, −*y*, −*z*+1.

### Hydrogen-bond geometry (Å, °)

**Table:**

*D*—H···*A*	*D*—H	H···*A*	*D*···*A*	*D*—H···*A*
C18—H18B···S1^ii^	0.99	2.99	3.749 (4)	134
C7—H7A···Cg1^iii^	0.99	2.67	3.567 (16)	151

Symmetry codes: (ii) *x*+1, *y*, *z*; (iii) *x*−1, *y*, *z*.
